# Special Issue “p53—Oncogene, Tumor Suppressor Gene, Guardian of the Genome and the Cell”

**DOI:** 10.3390/ijms27188233

**Published:** 2026-09-16

**Authors:** Dennis Rangno, Przemyslaw Bozko

**Affiliations:** 1 Department of Internal Medicine I, University Hospital Tübingen, 72076 Tübingen, Germany; 2 The M3 Research Center, Eberhard-Karls Universität Tübingen, 72076 Tübingen, Germany

More than four decades after its discovery, p53 remains a central regulator of cellular homeostasis and one of the most extensively studied proteins in cancer biology. Its designation as the “guardian of the genome” reflects its fundamental role in protecting cells from the consequences of genomic damage [1,2]. p53 functions as a central component of cellular stress response and can be activated by diverse stimuli, including DNA damage, oncogene activation, hypoxia, oxidative stress, and metabolic perturbations [3,4,5]. Depending on the nature and intensity of stress and the cellular context, p53 can promote cell-cycle arrest, DNA repair, senescence, apoptosis, or alternative adaptive responses [6,7]. These diverse outcomes contribute to its fundamental role as a barrier to malignant transformation.

The biological importance of p53 is reflected in the remarkable diversity of mechanisms through which its activity is disrupted in cancer. TP53 is the most frequently mutated tumor suppressor gene in human cancer, with mutations affecting close to half of human tumors [8,9]. However, disruption of the p53 pathway extends beyond direct TP53 mutation. In tumors retaining wild-type TP53, p53 activity can be attenuated through alterations in its regulatory network, including increased activity of the negative regulators MDM2 and MDM4 [10]. Conversely, many cancer-associated TP53 mutations not only abolish normal tumor-suppressive functions but they can also exert dominant-negative effects or confer oncogenic gain-of-function properties [11,12,13]. Mutant p53 proteins have been implicated in processes promoting cancer progression, including proliferation, invasion and metastasis, genomic instability, metabolic reprogramming, stemness, immune evasion, and remodeling of the tumor microenvironment [14]. Together with the increasingly recognized non-canonical functions of wild-type p53, these observations have shifted the field away from a simple model of p53 as a binary tumor suppressor toward a more dynamic and context-dependent view of the p53 network [15,16].

The recognition of p53 as a therapeutically actionable pathway also has a long history. Early approaches included adenovirus-mediated p53 gene replacement [17,18,19], while subsequent pharmacological studies demonstrated that p53 activity could be restored by targeting the p53–MDM2 interaction. The small molecule RITA was shown to bind p53 and inhibit its interaction with HDM2, resulting in restoration of p53 activity and induction of tumor-cell apoptosis [20]. In parallel, the development of the Nutlins provided a proof of principle that small molecules binding the p53-binding pocket of MDM2 could stabilize and activate wild-type p53 [21]. These studies helped establish pharmacological manipulation of the p53 pathway as a feasible therapeutic concept.

Since then, advances in structural biology, medicinal chemistry, and molecular pharmacology have substantially expanded the range of p53-directed therapeutic strategies. Current approaches include inhibition of MDM2- and MDM4-mediated suppression of wild-type p53, pharmacological restoration of mutant p53 activity, elimination or destabilization of mutant p53 proteins, gene-based approaches, and combinations with other anticancer treatments [22,23,24,25]. Despite considerable progress being made, translating these strategies into effective therapies remains challenging, in part because the consequences of TP53 alterations are highly heterogeneous and dependent on both the specific molecular alteration and the cellular context [12,23].

Several fundamental questions therefore remain unresolved. The functional consequences of many TP53 variants are still uncertain, the biological and clinical significance of individual p53 isoforms remains incompletely defined, and TP53 mutation status alone does not necessarily capture the functional state of the p53 pathway. Moreover, the expanding spectrum of non-canonical p53 functions raises important questions about how cellular and tissue context determines the biological outcome of p53 activity. Resolving these issues will be important not only for understanding p53 biology but also for developing more informative biomarkers and identifying tumors most likely to benefit from p53-directed therapeutic strategies.

Against this broader background, the contributions collected in this Special Issue, entitled “p53—Oncogene, Tumor Suppressor Gene, Guardian of the Genome and the Cell”, address complementary aspects of p53 biology, from fundamental mechanisms of its regulation and non-canonical functions to genetic and isoform diversity, clinical significance, and emerging therapeutic opportunities.

A natural starting point for this Special Issue is the well-established role of p53 in cell-cycle control, one of the fundamental mechanisms underlying its tumor-suppressive activity. In the original research article, “The Tumor Suppressor p53 Downregulates p107 (RBL1) Through p21–RB/E2F Signaling and Tandem E2F Sites”, Azzahrani and Alqahtani (Contribution 1) investigate the p53-dependent transcriptional regulation of RBL1, which encodes p107, a member of the retinoblastoma protein family. The authors demonstrate that p53 represses RBL1 expression indirectly through the p21–RB–E2F pathway and identify two conserved E2F-binding sites in the RBL1 promoter as essential elements of this regulation. Using p21-, RB-, and LIN37-deficient cells together with promoter assays, chromatin immunoprecipitation, and DNA pull-down experiments, they further show that the RB/E2F repressive complex plays the predominant role in this process, with additional contributions from other RB-family and E2F components. These findings provide a more detailed mechanistic understanding of the interplay between the p53 and RB/E2F tumor suppressor pathways in the control of cell-cycle progression.

While cell-cycle control represents one of the best-established functions of p53, the contributions to this Special Issue also illustrate how its biological role extends beyond canonical stress responses. In the Opinion, “Latest News from the ‘Guardian’: p53 Directly Activates Asymmetric Stem Cell Division Regulators”, Ana Carmena (Contribution 2) discusses a recently identified role of p53 in the regulation of asymmetric stem-cell division. Drawing on findings obtained in Drosophila neural stem cells, the article highlights the ability of p53 to transcriptionally activate key cell-fate determinants, including Numb and Brat. Interestingly, p53-mutant neural stem-cell lineages do not show tumor-like overgrowth despite reduced levels of these determinants, which the author attributes to substantial redundancy and compensatory mechanisms within the asymmetric cell-division machinery. The conservation of several components of this regulatory system further raises the question of whether similar mechanisms operate in mammalian cells, providing an interesting direction for future studies of p53 function in stem-cell biology and tumorigenesis.

The involvement of p53 in cellular processes extending beyond its canonical tumor-suppressive functions is further illustrated by its role in the regulation of inflammatory signaling. In the original research article, “MK2/p38/p53 Suppress Basal IL-1β and Non-Canonical NF-κB Signaling in Macrophages”, Herr et al. (Contribution 3) investigate the interplay between p53, the MK2/p38α signaling axis, and non-canonical NF-κB signaling in the regulation of IL-1β production. The authors show that the loss of MK2 or p38α in macrophages is associated with reduced basal p53 protein levels, activation of the non-canonical NF-κB pathway, and increased IL-1β expression. Mechanistically, p53 promotes caspase-3-dependent cleavage of RelB, thereby suppressing non-canonical NF-κB signaling and reducing the expression of IL-1β and TP53. These findings reveal an autoregulatory mechanism of p53 expression and provide an additional molecular link between p53 signaling, inflammation, and cancer.

Having considered the expanding spectrum of p53 functions, it is equally important to understand how alterations in the TP53 sequence may affect the molecular properties of the protein. In the original research article, “GnomAD Missense Variants of Uncertain Significance: Implications for p53 Stability and Phosphorylation”, García-Ayala et al. (Contribution 4) investigate 33 missense variants of uncertain significance (VUSs) reported in the gnomAD database using in silico approaches. Five variants were predicted to disrupt known phosphorylation sites, another five to create new consensus sequences for phosphorylation, and twenty to exert a moderate destabilizing effect on p53 structure. The authors identify p.S9N, p.S15N, and p.F338L as variants that may compromise p53 function. These findings illustrate the potential value of computational structural and functional analyses in assessing the possible impact of TP53 VUSs, while their biological and clinical significance remains to be established.

Beyond genetic variation, another source of functional diversity in the p53 system arises from the existence of multiple protein isoforms. In the review, “The p53 Isoforms as Potential Biomarkers in Different Cancer Entities”, Supina Pavić et al. (Contribution 5) provide an overview of the p53 isoform network, focusing on the expression profiles and biological functions of individual isoforms across different cancer types. Alternative promoter usage, alternative splicing, and alternative translation initiation generate multiple p53 isoforms that can interact with canonical full-length p53 and modulate its function. Their expression differs among tumor types and between malignant and normal tissues and has been associated with processes relevant to tumorigenesis, including apoptosis, cell-cycle regulation, migration, senescence, and stemness. The authors also discuss strategies for targeting p53 isoforms, either by altering the balance between individual isoforms or by targeting isoform-regulated downstream pathways. A better understanding of this regulatory layer may therefore contribute to the development of improved therapeutic approaches.

These different levels of p53 regulation raise the question of how reliably TP53 status or p53 protein abundance reflects the clinical behavior of a tumor. This issue is addressed in the original research article, “Proteomic Analysis of TCGA Data Reveals Limited Prognostic Value of p53 but Suggests BAX as a Potential Survival Marker in Cervical Carcinoma”, in which Toennießen-Klein et al. (Contribution 6) investigate the prognostic significance of TP53 mutation status, p53 protein levels, and selected downstream p53-regulated proteins in cervical carcinoma. Using clinical and proteomic data from 162 patients available through The Cancer Genome Atlas, the authors found that approximately 93% of tumors harbored wild-type TP53 and that TP53 mutation status was not associated with patient survival. Similarly, among tumors with wild-type TP53, p53 protein abundance showed no significant association with overall survival. p21 showed no prognostic relevance, while high TIGAR levels displayed only a slight, non-significant trend toward poorer survival. In contrast, elevated BAX levels showed a tendency to be associated with poorer overall and progression-free survival. Although this observation requires validation in larger cohorts, the study suggests that selected downstream effectors of p53 signaling may provide prognostic information not captured by TP53 mutation status or p53 protein abundance alone.

Beyond its biological and prognostic significance, p53 also remains an important focus of efforts to develop new therapeutic strategies. In the Commentary, “Harnessing p53 for Proximity Killing”, Zawacka (Contribution 7) discusses induced-proximity approaches for exploiting both wild-type and mutant p53 in cancer therapy. Particular attention is given to Regulated Induced Proximity Targeting Chimeras (RIPTACs), which can exploit the high intracellular abundance of mutant p53 as a tumor-selective scaffold. The Commentary highlights a proof-of-concept strategy in which a bifunctional molecule brings mutant p53 into proximity with the essential mitotic kinase PLK1, resulting in selective inhibition of TP53-mutant cancer cells without reactivation of mutant p53 to a wild-type state. Zawacka also discusses triple-action proteolysis-targeting chimeras (TAPTACs), which combine inhibition of MDM2 and MDM4/MDMX with BRD4 degradation, thereby restoring wild-type p53 activity while simultaneously targeting an oncogenic regulator. Together, these approaches illustrate how distinct molecular properties of wild-type and mutant p53 may be exploited in emerging proximity-based therapeutic strategies.

Taken together, the contributions to this Special Issue illustrate the remarkable breadth of p53 biology, extending from the regulation of fundamental cellular processes to its involvement in inflammation, cancer development, prognosis, and therapeutic targeting. They also emphasize that p53 function cannot be understood solely in terms of TP53 mutation status, but rather reflects multiple levels of regulation and is strongly influenced by the cellular and disease context. Despite more than four decades of intensive research, many aspects of p53 biology remain to be fully elucidated. Continued investigation of this remarkable protein and its regulatory network will further define how p53 can act as a guardian of cellular integrity, a tumor suppressor, or, under particular conditions, a contributor to oncogenic processes, while opening new opportunities for cancer diagnosis and therapy.
